# The parameter sensitivity of random forests

**DOI:** 10.1186/s12859-016-1228-x

**Published:** 2016-09-01

**Authors:** Barbara F.F. Huang, Paul C. Boutros

**Affiliations:** 1 Informatics and Bio-computing Program, Ontario Institute for Cancer Research, Toronto, Canada; 2 Department of Medical Biophysics, University of Toronto, Toronto, Canada; 3 Department of Pharmacology and Toxicology, University of Toronto, Toronto, Canada; 4 MaRS Centre, 661 University Avenue, Suite 510, Toronto, Ontario M5G 0A3 Canada

**Keywords:** Machine-learning, Random forest, Parameterization, Computational biology, Ensemble methods, Optimization, Microarray, SeqControl

## Abstract

**Background:**

The Random Forest (RF) algorithm for supervised machine learning is an ensemble learning method widely used in science and many other fields. Its popularity has been increasing, but relatively few studies address the parameter selection process: a critical step in model fitting. Due to numerous assertions regarding the performance reliability of the default parameters, many RF models are fit using these values. However there has not yet been a thorough examination of the parameter-sensitivity of RFs in computational genomic studies. We address this gap here.

**Results:**

We examined the effects of parameter selection on classification performance using the RF machine learning algorithm on two biological datasets with distinct *p/n* ratios: sequencing summary statistics (low *p/n*) and microarray-derived data (high *p/n*). Here, *p,* refers to the number of variables and, *n*, the number of samples. Our findings demonstrate that parameterization is highly correlated with prediction accuracy and variable importance measures (VIMs). Further, we demonstrate that different parameters are critical in tuning different datasets, and that parameter-optimization significantly enhances upon the default parameters.

**Conclusions:**

Parameter performance demonstrated wide variability on both low and high *p/n* data. Therefore, there is significant benefit to be gained by model tuning RFs away from their default parameter settings.

**Electronic supplementary material:**

The online version of this article (doi:10.1186/s12859-016-1228-x) contains supplementary material, which is available to authorized users.

## Background

Machine learning (ML) techniques are widely used in the analysis of high-throughput data to answer a broad range of biological questions. Applications in the field of medicine have transformed our understanding of complex genomic interactions and measurements [1]. ML has been successfully applied to biological disciplines including proteomics [2, 3], drug development [4, 5], DNA sequence analysis [6–8], cancer classification [9–13], clinical decision making [14, 15], and biomarker discovery [16, 17]. The versatility of ML algorithms to broad ranges of data and applications offers powerful, yet generalizable solutions to biological questions.

Recently, the random forest (RF) algorithm [18] for ML has achieved broad popularity. RF is a form of ensemble learning and possesses several characteristics that impart versatility. It can be applied to two-class or multi-class prediction problems, model interactions among variables, can take on a mixture of categorical and continuous variables, provides variable importance measures (VIMs), and has good predictive performance even for data with more variables (*p*) than samples (*n*; i.e. *p* > > *n*); potentially involving highly noisy and significantly correlated variables [19, 20]. Due to their non-parametric nature, RFs are fairly robust with relatively straightforward applications for inexperienced users [21, 22]. Consequently, this algorithm has expanded to a framework of models [23].

To train a random forest model, a bootstrap [24] sample is drawn, with the number of samples specified by the parameter *sampsize* [25]. By default, the bootstrap sample has the same number of samples as the original data: some samples are represented multiple times, whereas others are absent, leading to approximately 37 % of samples being absent in any given tree. These are referred to as the out-of-bag (OOB) samples [26]. Independent of the *sampsize* setting, after each sample is drawn, a decision tree is created. In the most commonly-used implementation, fully-grown or unpruned decision trees are created [18]. The number of trees is denoted by the parameter *n*_*tree*_ [21]. This collection of models is known as bootstrap aggregation or bagging [27] and is commonly applied to high-variance and low-bias learners such as trees [28, 29]. Since individual trees are more prone to over-fitting than a collection of trees, an ensemble method has a significant advantage [27, 29]; however, this is limited by the correlation between the trees and can be mitigated by choosing a number of randomly selected input variables at each split of the tree. The number of random variables used at each split is denoted by the parameter *m*_*try*_. Of this subset of randomly selected variables, the one that forms the best split is selected [25, 30]. The best split is selected on the basis of a specific objective function, most typically maximization of the Gini coefficient or total gain in purity. This produces the most homogeneous groups and lowest OOB error [21]. Several empirical studies have shown the benefit of aggregating multiple trees to create a strong learner whereas, independently they would be considered unstable with lower classification accuracy [27, 31–34].

Machine learning algorithms frequently require estimation of model parameters and hyper-parameters, commonly through grid-searching [35]. Surprisingly, though, this is not common practice in the literature for RFs, where default values are often used as it is widely believed that this method is parameter-insensitive, or at least robust to changes from default parameter settings [36–38]. To test this assumption, we performed an exhaustive analysis of the parameter-sensitivity of RFs in two large, representative bioinformatics datasets. We show that our top performing tuned models were able to achieve greater prediction accuracies than the default models for both datasets and that the performance of the default parameterization is inconsistent. This emphasizes the value of per-dataset tuning of RF models.

## Results

### Experimental design

To evaluate the sensitivity of RF models to parameterization, we selected two datasets representative of those commonly used in computational biology. The first studies quality-control metrics in next-generation sequencing [6] and comprises 15 features (sequencing quality metrics) with 720 training samples and 576 validation samples, and thus reflects low *p*/*n* ratio studies. Each sample was classified as “good library” or “bad library” based on information external to the 15 features, and our models aimed to predict this binary response variable.

The second dataset reflected high *p*/*n* studies and comprises three categorical clinical variables and 12,135 continuous mRNA abundances for Non-Small Cell Lung Cancer (NSCLC) patients [13]. We trained models to predict patient outcome, “no death” or “death”. There were 255 samples in the training cohort.

**Fig. 1:**
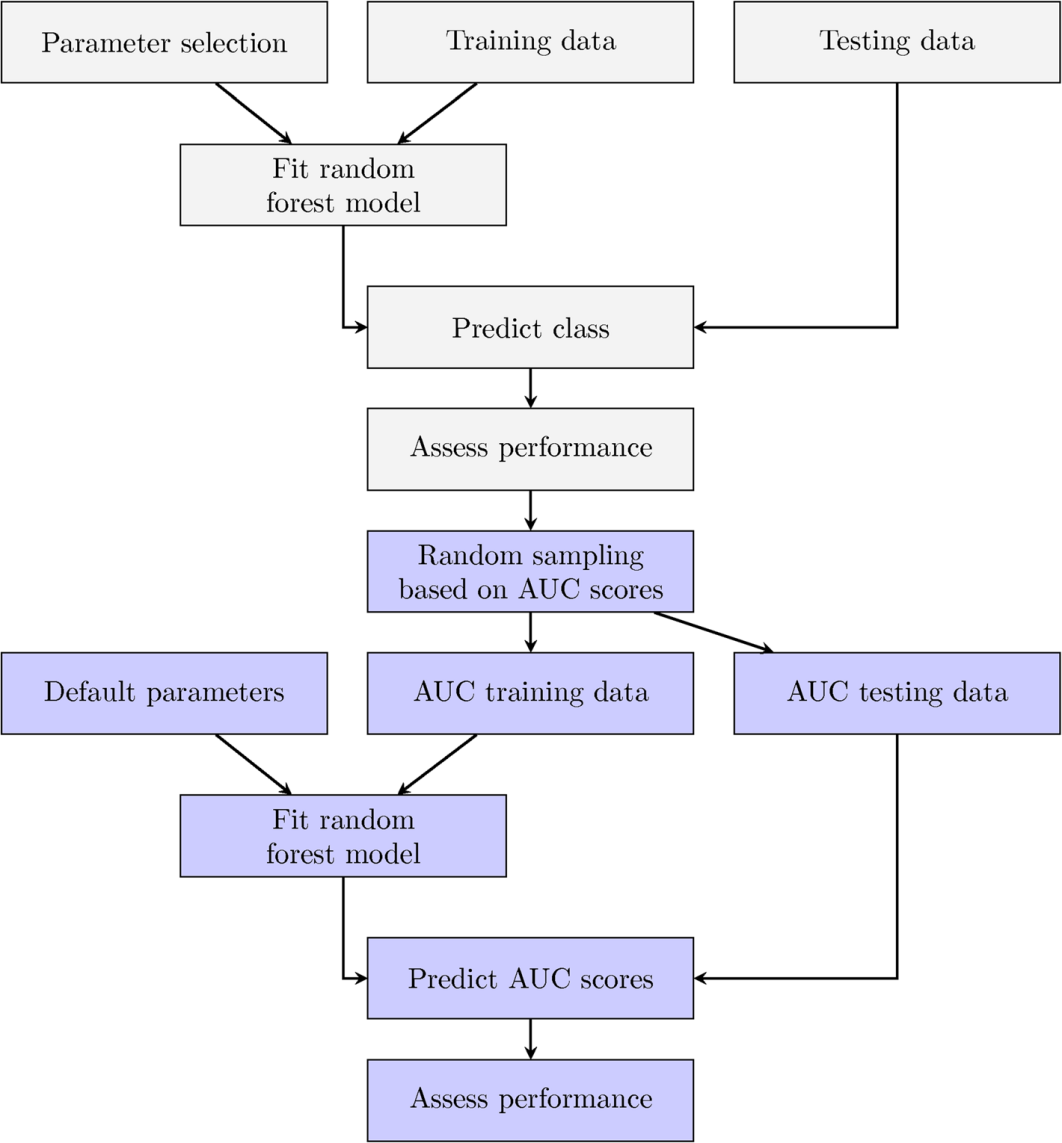
Experimental Design. Classification-based model fitting began with a unique combination of *n*_*tree*_, *m*_*try*_, and *sampsize* parameters in conjunction with training data, illustrated by the *gray* boxes. Each learned random forest model was used to predict the class of the validation data. Subsequently, AUC scores were calculated using the true class labels and these values were randomly subsetted into training and validation groups using 2/3 and 1/3 of the samples, respectively. In the second model fitting step, we evaluated whether AUC could be predicted from parameter sets alone. A RF regression model was fit using the parameters *n*_*tree*_, *m*_*try*_, and *sampsize* as variables and AUC as the response, illustrated by the *blue* boxes. Default settings were selected to train the RF regression models and AUC scores were predicted for the validation data. We evaluated the results using Spearman's and Lin’s correlation and determined the relative importance of each variable

For both datasets, we performed two model-fitting steps (Fig. 1). First, we selected a broad and comprehensive range of parameters (Additional file 1), and trained a RF classification model for each combination, including the default parameters. Models were trained on the training dataset and validated on a fully independent dataset. Performance was scored using the Area Under the Receiver Operating Characteristic Curve (AUC) [38]. Second, we fit an RF regression model using the data from the previous step: parameters were set as the covariates and AUC as the response. This allows us to characterize the association between prediction accuracy and parameterization. We randomly sampled 2/3 of parameter sets for training and reserved the remainder for validation. We aimed to predict the withheld AUC scores and assessed performance using Spearman's Rank Correlation Coefficient (*ρ*) and Lin's Concordance Correlation Coefficient (*ρ*_*c*_).

### Prediction accuracy is a strong function of parameterization in low *p/n* studies

**Fig. 2:**
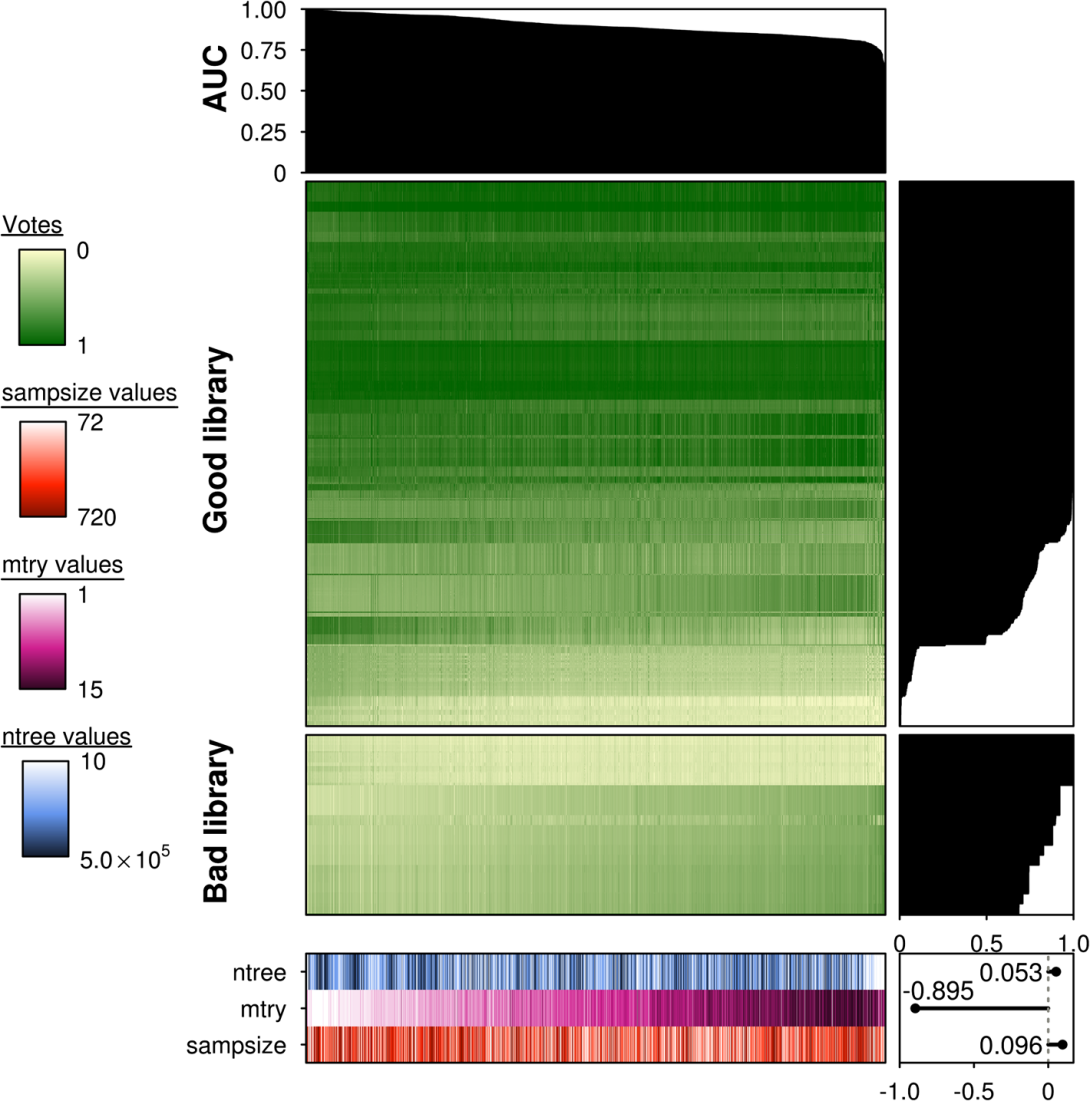
Prediction accuracy is a strong function of parameterization in low *p/n* studies. Summary of low *p/n* predicted votes for each fitted random forest model (*n* = 1500). An AUC plot is provided at the top indicating the relative performance of each model, represented by each column. Each model was fitted from a unique combination of *n*_*tree*_ (*n* = 10), *m*_*try*_ (*n* = 15) and *sampsize* parameters (*n* = 10) and their respectively outcomes (votes) for each sample or row (*n* = 576). Votes are provided in values from 0–1 with 0 representing a “bad library” and 1 representing a “good library”. All columns are ordered in descending order of AUC scores and rows are ordered in descending order of the fraction of correct votes for a given sample (total votes for the true sample class/all votes). All samples were subsetted according to the true class labels “good library” and “bad library”, though the votes may not be reflective of this. Barplots for vote fractions are provided on the right of the main heatmaps and the values for each parameter are provided at the bottom of the figure. The *n*_*tree*_ parameter is illustrated in *blue*, *m*_*try*_ in *magenta* and *sampsize* in *orange*. Lighter hues represent lower values with darker hues indicating higher values. A scatterplot in the bottom right corner illustrates a strong negative correlation between the *m*_*try*_ parameter with AUC scores (*ρ* = -0.89, *p =* 0)

We first evaluated the parameter sensitivity of RF prediction accuracy in the low *p*/*n* dataset. We created 1,500 different sets of parameters and evaluated the performance of each. Most models succeeded at this task (Fig. 2), with a median AUC of 0.893 and 96 % of models exceeding 0.80 AUC. However, the performance varied dramatically, with a range of 0.6113–0.9996, suggesting that some parameterizations greatly improve or hinder prediction accuracy. The default parameterization (*n*_*tree*_ = 500, *m*_*try*_ = 3, *sampsize* = 720 with replacement) performed well, with an AUC of 0.9726 and ranked in the top 12 % of all models (174/1,500; Additional file 2). This clearly demonstrates that the default settings are reasonable, but not optimal.

We asked if models were consistently struggling with the same samples. We looked for samples in the validation dataset where at least 50 % of models trained with different parameter sets made incorrect predictions. In total 73/576 (12.7 %) of validation samples were difficult to classify. These were strongly asymmetrically distributed between the classes with 72/432 (17 %) “good library” validation samples difficult to classify relative to only 1/144 (1 %) “bad library” validation samples (*p* = 1.27 × 10^−6^; proportion-test). Interestingly though, the global error rate was not dramatically different between these two groups (20 % for “good library” vs. 14 % for “bad library” samples).

Parameterization was strongly correlated to AUC score (Fig. 2) in this dataset, but tightly focused on specific parameters. The number of variables sampled per node (*m*_*try*_) was strongly negatively correlated with AUC (*ρ*_*mtry*_ = -0.895) and *m*_*try*_ ≤ 3 resulted in higher classification accuracy (mean AUC for *m*_*try*_ ≤ 3 = 0.97; mean AUC for *m*_*try*_ > 3 = 0.88; Welch Two Sample *t*-test). In contrast, models were relatively robust to changes in the *n*_*tree*_ and *sampsize* parameters (*ρ*_*ntree*_ = 0.053 and *ρ*_*sampsize*_ = 0.096; Spearman's *ρ*).

To further explore the relationship between parameterization and performance, we univariately compared performance within each parameter (Additional file 1), with Benjamini-Hochberg adjustment for multiple-testing [39]. While *sampsize* values did not differ significantly from each other, however, *n*_*tree*_ of 10 had significantly lower AUCs (*q* < 0.05) than other setting (Additional files 3, 4 and 5). Similarly, as noted above there was a near-linear relationship between increasing *m*_*try*_ and decreasing AUC in the validation cohort (Additional file 6). These findings illustrated the strong influence of parameter selection on classification accuracy, and that both linear and threshold effects can be observed.

While the results to this point demonstrate both that parameterization powerfully influences prediction accuracy and that the default parameter settings are sub-optimal. However they do not demonstrate if it is possible to improve upon the defaults via parameter-optimization studies. We therefore implemented 10-fold and stratified 10-fold cross-validation using the parameters in Additional file 1. The data was randomly divided into 10 even folds, using 9/10 folds for training and the last fold for validation. This step was repeated so that each fold was used for validation once, so that the number of samples in validation was equal to the number of samples in the original training set (*n* = 720). All validation folds were pooled to evaluate AUC and cross-validated models were compared to non-cross-validated models using Spearman's *ρ* and Lin's *ρ*_*c*_ (Additional file 2).

Predicted classes for both 10-fold cross-validation and stratified 10-fold cross-validation were weakly, but statistically-significantly correlated to the predicted classes for non-cross-validated results (Additional file 7a-b), and strongly correlated to one another (Additional file 7c).

We found that cross-validation and stratified cross-validation resulted in 97 % of models having an AUC of 1, including the defaults. We used an additional metric, root mean squared error (RMSE) to break ties. The optimal model in 10-fold cross-validation (rank = 1, *n*_*tree*_ = 500000, *m*_*try*_ = 10, *sampsize* = 720) had a RMSE of 0.00203, whereas the default model (rank = 579) had a RMSE of 0.0273. The optimal model in stratified 10-fold cross-validation (rank = 1, *n*_*tree*_ = 50, *m*_*try*_ = 14, *sampsize* = 648) had a RMSE of 0.0119, whereas the default model (rank = 319) had a RMSE of 0.0229. Overall, we found that 39 % (578/1500) and 21 % (318/1500) of models outperformed the untuned model (*n*_*tree*_ = 500, *m*_*try*_ = 3, *sampsize* = 720), respectively. Twenty one percent (310/1500) of these models shared the same parameter values and were found to perform better than the default settings in both cross-validated and non-cross-validated results. We found the addition of a second metric, RMSE useful in breaking ties and assessing model performance for low *p/n* data.

### Prediction accuracy can be a strong function of parameterization in high *p/n* studies

**Fig. 3:**
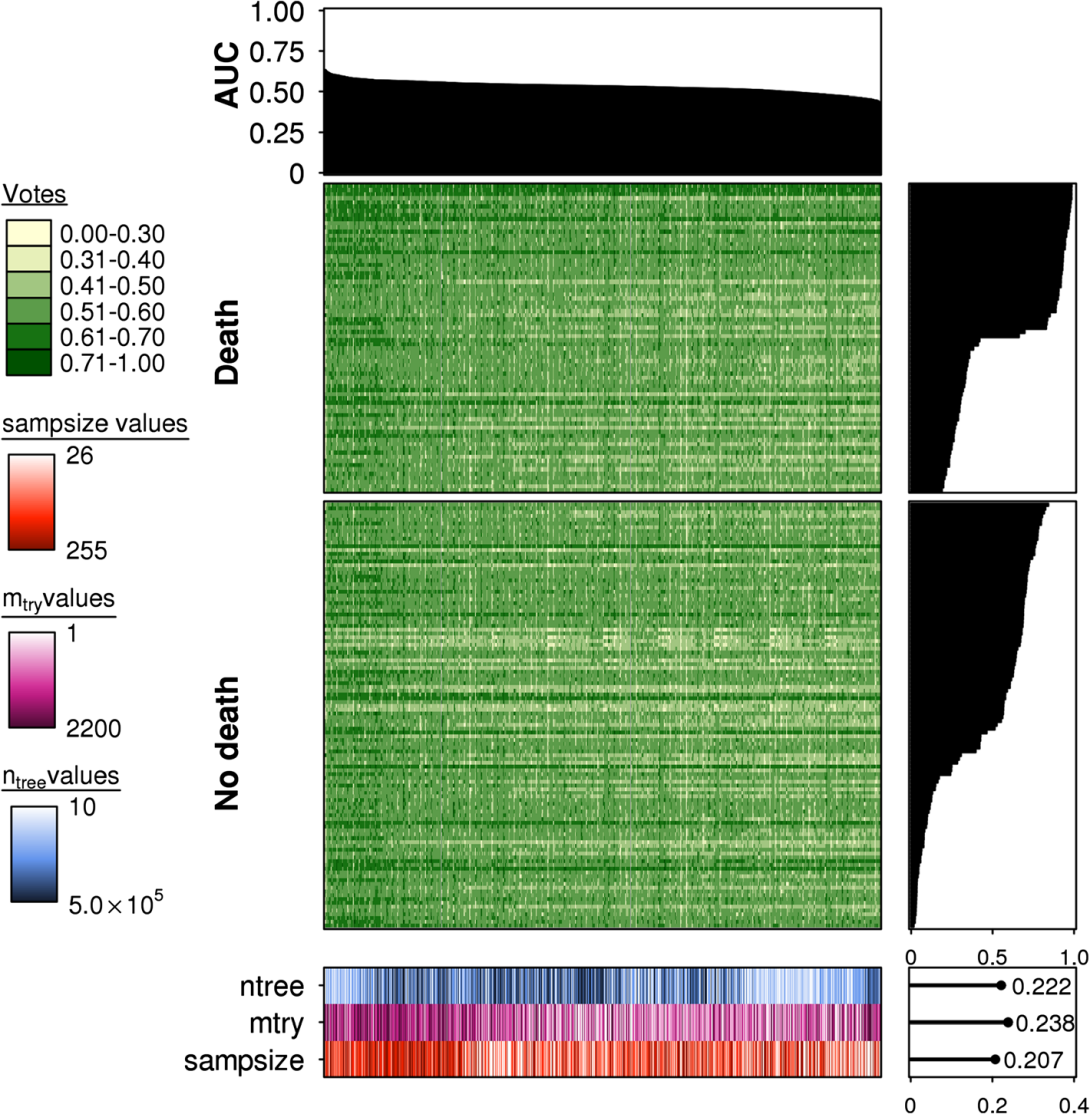
Prediction accuracy is a strong function of parameterization in high *p/n* studies. Summary of the predicted votes for the combined validation data for each fitted random forest model (*n* = 1000). A barplot for AUC scores is provided at the top indicating the relative performance of each model, represented by each column. Each model was fitted from a unique combination of *n*_*tree*_ (*n* = 10), *m*_*try*_ (*n* = 10) and *sampsize* parameters (*n* = 10) and their respectively outcomes (votes) for each sample or row (*n* = 186). Votes are provided in values from 0–1 with 0 representing a “no death” event and 1 representing a “death” event. All columns are ordered in descending order of AUC scores and rows are ordered in descending order of the fraction of correct votes for a given sample (total votes for the true sample class/all votes). All samples were subsetted according to the true class labels “death” and “no death”, though the votes may not be reflective of this. On the right of the main heatmaps are respective barplots for vote fractions and a heatmap of parameter values is present at the bottom of the figure. The *n*_*tree*_ parameter is illustrated in *blue*, *m*_*try*_ in *magenta* and *sampsize* in *orange*. Lighter hues represent lower values with darker hues indicating higher values. To the right of this is a scatterplot illustrating Spearman's correlations of each parameter with the AUC scores; positive correlations were observed for the parameters *n*_*tree*_, *m*_*try*_, and *sampsize* (*ρ* = 0.222, *p* < 10^−10^; *ρ* = 0.238, *p* < 10^−12^; *ρ* = 0.207, *p* < 10^−9^, respectively)

To contrast these data, we examined the effects of parameterization on prediction accuracy for high *p/n* data [13] (Additional file 1). We created 1,000 different sets of parameters and evaluated the performance of each (Additional file 8). Again, we saw that model performance varied greatly with parameterization with a median AUC of 0.533 and 2 % of models exceeding an AUC of 0.60 (Fig. 3). However, the performance varied dramatically, with a range of 0.4254–0.6337, suggesting that some parameterizations could greatly improve or hinder prediction accuracy. The default parameterization (*n*_*tree*_ = 500, *m*_*try*_ = 110, *sampsize* = 255) performed well relative to other models, with an AUC of 0.6098 and ranked 10th. This demonstrates the near optimal performance of the default settings.

We asked if models were consistently struggling with the same samples. We looked for samples in the validation dataset where at least 50 % of models trained with different parameter sets made incorrect predictions. In total 89/186 (48 %) of validation samples were difficult to classify. These were symmetrically distributed between the classes with 37/74 (50 %) “death” events difficult to classify compared to 52/112 (46 %) “no death” samples (*p* = 0.74; proportion-test). The error rate was significantly different between these two groups (for “no death” samples; *p* = 0; proportion-test).

Parameterization was strongly correlated to AUC in this dataset, with contribution from all parameters. We observed that *m*_*try*_ (*ρ* = 0.238, *p* = 2.12 × 10^−14^; Spearman’s correlation) was the most correlated, followed by *n*_*tree*_ (*ρ* = 0.222, *p* = 1.39 × 10^−12^; Spearman’s correlation) and *sampsize* (*ρ* = 0.207, *p* = 3.73 × 10^−11^; Spearman’s correlation).

To further explore the relationship between parameterization and performance, we univariately compared performance within each parameter (Additional file 1), with Benjamini-Hochberg adjustment for multiple-testing [39]. We observed that larger *n*_*tree*_ values resulted in higher prediction accuracy and reduced performance variability compared to lower values (*q* < 10^−8^), with no significant difference observed between values *n*_*tree*_ ≥ 10,000 (Additional files 9 and 10). Similar results were observed for *sampsize* and *m*_*try*_ (Additional files 11 and 12) where there was a near-linear relationship between increasing parameter values and AUC in the validation cohort. Additionally, no significant differences were observed in AUC for *sampsize* ≥ 153 and *m*_*try*_ ≥ 110. The *m*_*try*_ value here is notable since it was used as the default, providing some support to previous claims that the default performs well. These findings illustrated the strong influence of parameter selection on classification accuracy, and that both linear and threshold effects can be observed.

### Parameters can be used to predict performance

Having shown that model performance is strongly influenced by *n*_*tree*_, *m*_*try*_, and *sampsize*, we next asked how strongly these three parameters could predict AUC directly. We assessed variable importance using the Gini VIM, where larger values indicate a variable is more important for accurate classification. We were able to predict AUCs using this metric that closely reflects those of the true data for low *p/n* data (Additional file 13a; *ρ* = 0.92, *p* = 1.29 × 10^−209^, *ρ*_*c*_ = 0.89; Spearman's *ρ* and Lin's *ρ*_*c*_). We observed that *m*_*try*_ demonstrated the highest Gini VIM for low *p/n* data (Additional file 13b).

Similar results were observed for the high *p/n* data, where prediction accuracy was a strong function of parameter selection across all validation sets (Additional file 14a; *ρ* = 0.48, *p* = 5.42 × 10^−21^, *ρ*_*c*_ = 0.33; Spearman's *ρ* and Lin's *ρ*_*c*_). Interestingly, the parameters demonstrated relatively balanced importance measures with *sampsize* demonstrating the highest Gini VIM and *n*_*tree*_ with the lowest (Additional file 14b).

### Importance ranks can be sensitive to parameter changes

Finally, we asked if parameterization change could alter the identification of importance variables (which are frequently used in feature-selection approaches, for example) [23, 36]. We focused on the low *p/n* data, and trained models using the settings in Additional file 1 and ranked permutation VIM for each quality metric from 1–15, with 1 representing the most important variable. Permutation VIM is the mean decrease in classification accuracy after a random variable is removed from model fitting. Larger values suggest a variable has more discriminative power [40, 41].

**Fig. 4:**
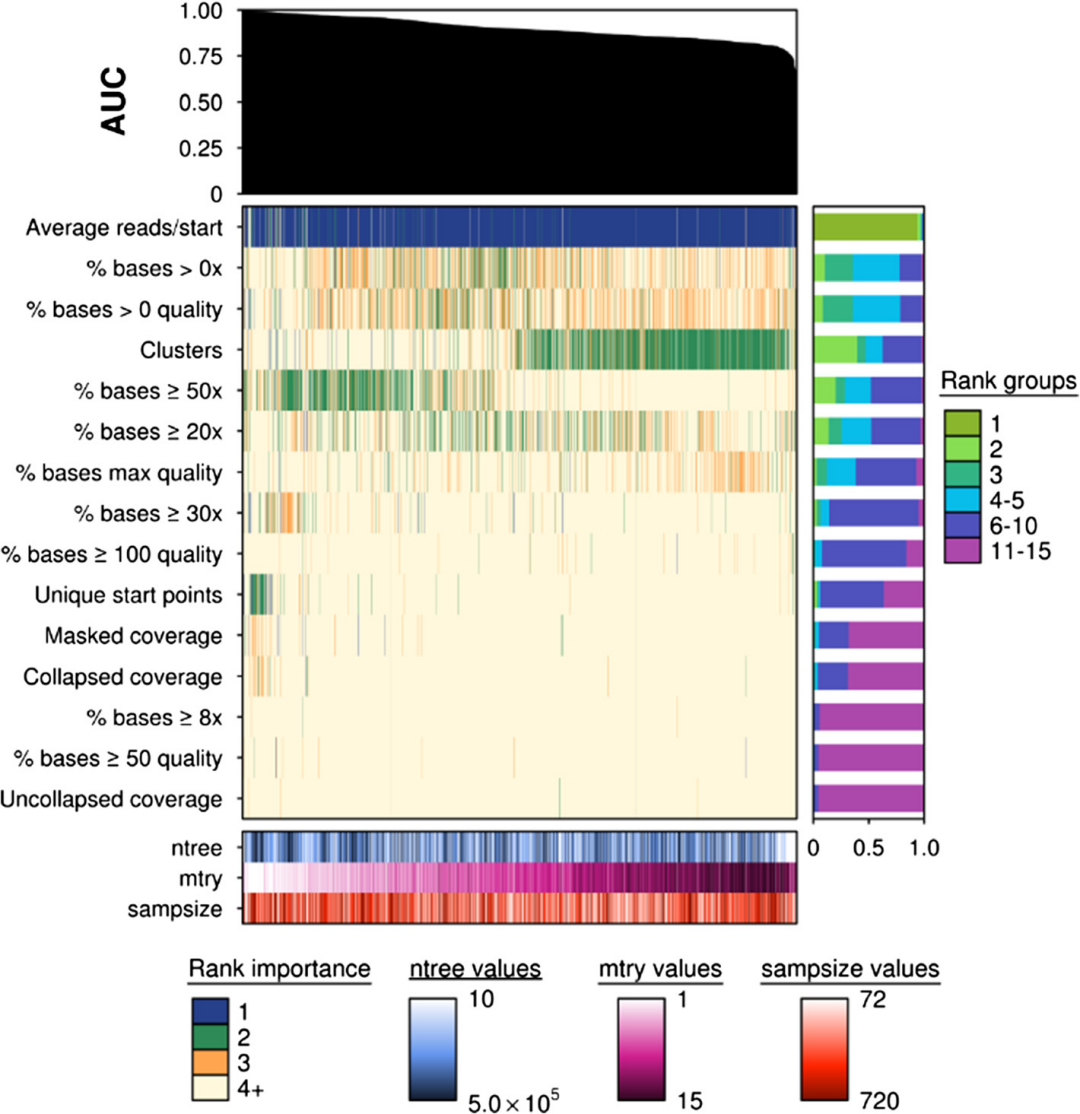
Importance ranks can be sensitive to parameter changes in low *p/n* studies. Summary of the variable importance ranks for each sequencing metric (*n* = 15). An AUC plot is provided at the top indicating the relative performance of each model, represented by each column. Each model was fitted from a unique combination of *n*_*tree*_ (*n* = 10), *m*_*try*_ (*n* = 15) and *sampsize* parameters (*n* = 10) and their respectively outcomes (importance value) for each metric. Each column of the main heatmap corresponds to a model's importance values, and were ranked from 1–15, where 1 represented the most important feature and 15 the least. The importance values were ordered according to previously calculated AUC scores using predicted vote and true class labels. Each row represents a metric and are ordered according to the mean rank of its importance values. The importance values were simplified in the main heatmap and illustrate four groups only. *Blue* indicates a rank of 1, *green* a rank of 2, *gold* a rank of 3, and *beige* a rank of 4 and greater. A summary of overall rank groups for a particular metric are illustrated in a barplot on the right of the main heatmap and a covariate heatmap with all parameter combinations is illustrated at the bottom of the plot. The *n*_*tree*_ parameter is illustrated in *blue*, *m*_*try*_ in *pink* and *orange* for *sampsize* in *orange*. Some parameters demonstrate robust behaviour to parameter changes such as “Uncollapsed coverage” and “% bases ≥ 50 quality”, which were ranked between 11–15 inclusive in 96 % and 95 % of all samples, respectively. These variables possessed VIMs that suggested they were less influential on classification accuracy. Yet, “Average reads/starts” was insensitive to parameter changes and was considered the most important variable. Another variable “Clusters” was parameter sensitive, illustrating that variables vary in their sensitivity to parameter changes which can ultimately influence classification accuracy

Variables differed in their sensitivity to parameter changes when evaluating variable importance (Fig. 4). The variable “Average reads/starts” was robust against parameter changes and was considered the most important in 94 % of all samples, whereas “Clusters” exemplified strong parameter sensitivity and was positively correlated to *m*_*try*_. On the other hand, “% bases ≥ 50 %” was found to have higher VIMs with lower *m*_*try*_ values.

Our order for variable importance deviated from that of the original study [6], where “% bases ≥ 8×” was reported as the most discriminative variable. We examined how variable importance changed with differing *n*_*tree*_ values (*n* = 10) while holding *m*_*try*_ and *sampsize* constant (*m*_*try*_ = 3, *sampsize* = 720; Additional file 15) and observed that larger *n*_*tree*_ values led to more stable VIMs.

## Discussion

There are two common assumptions regarding RF models. The first is that the default parameters lead to good performance [37, 38] and the second is that the algorithm is robust to parameter changes [19, 21, 42]. To help quantify the wide-spread nature of these assumptions we manually reviewed all papers published in BMC Bioinformatics between January 1, 2015 and November 21, 2015 (Additional file 16). We looked for papers that referenced the canonical RF paper [18] during this ~11 month period. Of the 16 papers that implemented RFs, exactly half performed a parameterization study to optimize parameters, and only 5/16 papers reported the final parameter setting used. That is, about half of RF-studies could benefit from improved parameterization and another third from improved reporting. This highlights clearly the gap between machine learning theory and practice, and gaps in methods reporting that are not being caught by peer-review.

Parameterization is difficult and its absence from the model fitting process may be due to limited experience, a lack of readily available heuristics or limited resources [43]. Consequently, these factors lead to the inappropriate selection of parameters or lack thereof, directly influencing learning [44]. We sought to determine the effects of parameterization on classification accuracy and variable importance measures. Our findings suggested data-dependent parameter sensitivities ultimately influence classification accuracy and VIMs for binary classification problems. Our findings may not extend to regression analyses or multi-class problems, where the relationship between the variables and response is much more complex.

We observed that the default parameters have the potential to perform well, however results across all tests indicated that parameter tuning enabled higher model performance. The majority of high performing parameter combinations did not coincide with general patterns observed in the pattern selection process i.e., in most samples higher parameter values led to greater classification accuracy and the top performing parameters had lower values. Such models may have performed well due to random chance or were over-fit. These results emphasize the importance of parameter tuning and how one cannot rely on any arbitrary parameter set to perform well. This also suggests that existing publications implementing untuned models may improve classification accuracy through model tuning. To reduce computation time and work for parameter selection, we applied a RF regression model, which predicted model performance more accurately than the more expensive 10-fold cross-validation and stratified 10-fold cross-validation. The RF regression model was also better at discriminating poor performing parameter sets from high performing parameter sets.

To our knowledge, this is the first computational genomic study that addresses parameter sensitivities using a comprehensive range of values for two unique biological data types. In particular, we observed that the low *p/n* data was sensitive to changes in *m*_*try*_ and the high *p/n* data demonstrated a synergism between all three parameters. Additionally, not all variables exhibited robust behaviour towards parameter changes when determining VIMs (e.g., “Clusters” and “% bases ≥ 50×”). These findings challenge the assumption that RFs are relatively robust. Parameters that did not a play key role independently had an observable and significant synergism when constructing RF regression models with interaction terms (from section Parameters can be used to predict performance).

We also noted that our variable importance ranks did not coincide with [6]. This was largely explained by the bias in feature importance for the RF algorithm. Variables that were highly correlated to truly influential variables or have more categories will be over-selected by the algorithm and do not reflect the true relative contribution of a variable in a classification or regression problem [20]. Chong et al. [6] implemented an alternate algorithm, “cforest”, from the R package “party” to generate unbiased VIMs. One area for future research is to investigate the sensitivity of parameter changes in the “cforest” algorithm.

Moreover, characteristics of the data, such as, *p > > n* and minor class imbalances were observed. The numerous variables in the high *p/n* data constrained the selection range of *m*_*try*_ parameters, potentially confounding the results. In such samples, *m*_*try*_ ≠ *p*. This was not the sample for the low *p/n* data, where we were able to test all possible values of *m*_*try*_. This limitation may also be viewed as beneficial since the number of randomly selected variables at each split is constrained and therefore, limits tree correlation within a forest.

An additional data characteristic limiting the classification accuracy in RF could be class imbalance [45, 46]. The unequal number of classes in a dataset is technically considered class imbalance, however, in the scientific community, class imbalance corresponds to data with significant to extreme disproportional class numbers, such as, 100:1 or 10,000:1 [47]. These types of “imbalanced data” were not considered here. Furthermore, the minor classes “bad library” and “death” in the small *p/n* data and high *p/n* data respectively, had a higher classification accuracy suggesting, in some instances, the heterogeneity of a sample is more influential on classification accuracy. We also aimed to mitigate class imbalance effects through stratified sampling and by using the AUC performance metric. Alternate methods such as, cost sensitive learning [48] and artificially balancing the data through down sampling the majority class [49], over sampling the minority class [50], or both [51] have been shown to deal with class imbalance effectively. Artificial balancing ensures that class priors are equal in tree classifiers and that the minority class is included in the bootstrap sample. On the other hand, cost sensitive learning incurs a greater cost for misclassified minority samples over majority samples. Minor class imbalances were not observed to be an issue in this study, however, data should be analysed with caution in highly imbalanced studies.

## Conclusions

We analysed the effects of parameterization using exhaustive selection methods and showed that tuning can be successfully applied to a non-parametric machine learning algorithm to improve prediction accuracy. Although we only examined two different genomic datasets, we observed that parameter sensitivities are data-specific, necessitating per-dataset tuning. Our findings illustrate this through discordant correlations between parameters and performance scores for low *p/n* and high *p/n* data. The model fitting process is a fundamental step in machine learning and careless parameter selection can lead to sub-optimal models and potentially missed findings.

## Methods

### Datasets

We explored parameterization of RFs on two datasets. The first was a sequencing-derived dataset (low *p/n* data) [6] and the second was a microarray-derived dataset (high *p/n* data) [13], reflecting low and high *p/n* data, respectively.

The low *p/n* data (15 variables with 1,296 samples) contained 15 quality metrics describing overall coverage, coverage distribution, basewise coverage and basewise quality of 53 whole genomes. The data was derived for the International Cancer Genome Consortium (ICGC) project to predict the amount of sequencing that is required to reach a given coverage depth for 1/8 lane samples [6]. The outcome column was a list of binary values (0 for “bad library” or 1 for “good library”) indicating whether the target coverage depth was reached (30× for normal, 50× for tumour). The data was split into training and validation sets, as described by the low *p/n* paper [6] and contained 720 and 576 samples, respectively.

The high *p/n* data contained gene expression data for 442 lung adenocarcinomas and basic clinical covariates (stage, age and sex) to predict lung cancer patient outcome (0 for “no death” or 1 for “death”). The data were collected from six contributing institutions and grouped into four subsets based on the laboratory where processed (University of Michigan Cancer Center (UM), Moffitt Cancer Center (HLM), Memorial Sloan-Kettering Cancer Center (MSKCC), and Dana-Farber Cancer Institute (DFCI)). All facilities processed the data using the same robust and reproducible protocol.

The first two datasets, UM and HLM, were grouped together to form the training set (12,138 variables with 255 samples), while the MSKCC data (104 samples) and DFCI data (82 samples) formed the validation set (186 samples).

### Parameter selection

The *m*_*try*_ parameter values were selected using factor levels of the default value. Since the nature of this supervised learning problem is that of classification and not regression, the default value of *m*_*try*_ is the square root of the number of variables or features in the data 18 √*p*, whereas, in regression the default is *p*/3. The study by [21] reported *m*_*try*_ as the most sensitive parameter with values of *m*_*try*_ factor = 1/2 (1/2• 18 √*p*), *m*_*try*_ factor = 1 (18 √*p*) and *m*_*try*_ factor = 2 (2•18√*p*) showing good performance. Given this information and the number of variables in the data, one to all variables were selected as *m*_*try*_ values for the SeqControl dataset (*p* = 1-15), the *m*_*try*_ values 1, 5, 11, 22, 55, 110, 220, 550, 1100, 2200 were selected for the NSCLC data (*p* = 12,138). The NSCLC values were obtained by selecting factor levels (1/100, 1/20, 1/10, 1/2, 1, 2, 5, 10, 20), multiplying them with *p* and taking the largest integer preceding a specified number i.e., for a value of 3.4, 3 was used.

The values for *n*_*tree*_ were selected similarly to those for *m*_*try*_. We imposed factor levels to the default value and took the product to create the *n*_*tree*_ values. The factor levels were 1/50, 1/10, 1/5, 4/10, 1, 2, 20, 100, 200 and 1000. The final *n*_*tree*_ values were 10, 50, 100, 200, 500, 1000, 1e4, 5e4, 1e5, 5e5. The selected *n*_*tree*_ values were the same for both datasets.

The final parameter *sampsize*, had the same factor levels for both datasets and was a sequence of values from 0.1–1, increasing by increments of 0.1. To obtain the final *sampsize* values, we multiplied the total number of samples in training by the *sampsize* factor levels and took the smallest integer proceeding a number i.e., for a value of 3.4, 4 was used.

Selected parameters were used to train models with the function “randomForest” using sampling with replacement. The data was partitioned according to the original papers, as described above. In the SeqControl data experiment, we aimed to predict whether the target of sequencing depth coverage was achieved using 1/8 lane (1 for “good library”, 0 for “bad library”). In the NSCLC data experiment, we aimed to predict patient outcome (1 for “death”, 0 for “no death”). A table of complete parameter settings for the SeqControl data and NSCLC data can be found in Additional file 1.

### Model training

The data were trained using the function “randomForest” from the R package “randomForest” (v4.6-10) [21, 52]. A series of RFs were trained on each dataset using a unique combination of the three parameters: *n*_*tree*_, *m*_*try*_ and *sampsize*. For the SeqControl data, we used 15 *m*_*try*_ values, 10 *n*_*tree*_ values, and 10 *sampsize* values. These values and numbers differed slightly in the NSCLC training: 10 *m*_*try*_ values, 10 *n*_*tree*_ values, and 10 *sampsize* values. A resulting total of 1500 and 1000 unique combination were obtained for model fitting on the SeqControl data and NSCLC data, respectively.

After training, each model was then validated on independent validation data to obtain class probabilities (votes). The votes and true class labels were then used to estimate model performance by calculating the AUC score.

### Performance prediction using parameters as variables

In order to determine whether model performance could be predicted, we performed regression using RF, on a subset of parameters and their respective AUC scores. AUC scores were calculated by comparing the predicted votes from each model to the true classifications. We initially attempted this from a linear model approach, however, classification accuracy was low due to overfitting. After subsetting 2/3 of the data into training and 1/3 for validation, we performed model tuning and selected the model with the lowest mean squared error. Tuning was conducted using a grid of parameters (Additional file 17) and 5-fold cross validation. We then applied the optimal settings (*n*_*tree*_ = 200, *m*_*try*_ = 2, *sampsize* = 200) to train a RF model. The response for our model was AUC score and the variables were *n*_*tree*_, *m*_*try*_ and *sampsize*. The expression for the model formula included the terms in an additive and interaction format i.e., *sampsize* + *m*_*try*_ + *n*_*tree*_ + sampsize*m_*try*_ + sampsize*n_*tree*_ + *n*_tree_* *m*_*try*_ + sampsize*n_tree_*m_*try*_. After training and validating the models, we were able to assess performance using the following metrics, Spearman's *ρ*, Spearman's *p*-value (*P*) and Lin's *ρ*_*c*_. Lastly, importance values were found for each variable (*n*_*tree*_, *m*_*try*_ or *sampsize*) in the form of Gini VIM.

### Model selection using 10-fold cross-validation and stratified 10-fold cross-validation

Ten-fold cross-validation was used to estimate the generalization error of each unique RF model (*n* = 1500) for the SeqControl data. This method of cross-validation has been suggested to perform better than the more expensive leave-one-out cross-validation [53]. The data was subsetted into 10 even folds, with nine groups selected for training and the last reserved for validation. This process was iterated until each fold was used in the validation stage once, so that the number of samples in validation was equal to the number of samples in the original training set (*n* = 720).

The above was repeated for stratified 10-fold cross-validation with an even distribution of the minority class among each fold. A total of 72 samples appeared in each fold with approximately 14 samples of the minority class and 58 of the majority class. AUC scores were used to estimate accuracy and correlations were calculated between non-cross-validation, 10-fold cross-validation and stratified 10-fold cross-validation results. A table comparing the above three methods is in Additional file 2.

### Ranking variable importance

Additional information pertaining to variable importance was collected from training and validating the SeqControl models using permutation VIM [54]. Permutation VIM can be interpreted as the mean decrease in accuracy of a RF due to the removal of a variable. The magnitude of the value is directly proportional to the relative contribution of a particular variable in classifying samples, that is, the greater the decrease or drop in accuracy, the more a feature is correlated to the response.

The model for the SeqControl data had additional settings that were implemented, such as “importance”, “localImp”, “proximity” and “keep.inbag”. These arguments were all set to “TRUE” to keep results relatively consistent with the original paper [6].

Due to the exhaustive parameter selection method of grid searching, we parallelized jobs using Perl High Performance Computing Interface (HPCI) [55] and parallelized jobs further by using the R package, “foreach” (v1.4.2) [56].

### Statistical model evaluation

We evaluated the performance of models using several statistical measures in the R statistical environment (v3.1.3) [57]. For classification accuracy, we calculated the AUC using the predicted votes and the true class labels with the function “auc” from the package pROC (v1.8) [58]. For non-parametric tests comparing the parameter performance in classification, we used the function “cor” from the base “stats” package (v3.2.0) [57] to calculate Spearman's *ρ* and to find the correlation coefficient between the AUC scores and the parameter of interest. Spearman's *ρ*, Spearman's *p*-value and the equation for Lin's *ρ*_*c*_ from the paper [59] were used to determine the correlation between true and predicted AUC values in performance prediction. All *p*-values were adjusted using the function “p.adjust” from the base “stats” package (v3.2.0), using the Benjamini-Hochberg procedure.

### Data visualization

Figures were generated in the programming language LaTeX and in the R statistical environment (v3.1.3) using custom R scripts for the “lattice” (v0.2-31) [60] and “latticeExtra” (v0.6-26) [61] packages.

## Additional files

Additional file 1: RF parameter settings. RF parameter settings for low *p/n* data (SeqControl; *p* = 15) and high *p/n* data (NSCLC; *p* = 12,138). (CSV 515 bytes) Additional file 2: AUC results for low *p/n* data. Low *p/n* results for prediction accuracy using AUC as the performance metric for non-cross-validation results, 10-fold cross-validation and stratified 10-fold cross-validation. Ranks indicate the relative performance of different models with lower ranks representing higher performing models i.e., a rank of 1 is the best model. The default settings (*n*_*tree*_ = 500, *m*_*try*_ = 3, *sampsize* = 720) are found on row 1502 of the table. (CSV 116 kb) Additional file 3: Pairwise *t*-test results for *sampsize* intra-parameter groups for the low *p/n* data. All *p*-values were adjusted using a Benjamini-Hochberg procedure. There were no groups that differed significantly from each other. (TXT 333 bytes) Additional file 4: Pairwise *t*-test results for *n*_*tree*_ intra-parameter groups for the low *p/n* data. All *p*-values were adjusted using a Benjamini-Hochberg procedure. The only *n*_*tree*_ value found to differ from every other *n*_*tree*_ group was 10. (TXT 456 bytes) Additional file 5: Intra-parameter values display variation in low *p/n* studies. We evaluated the parameters *sampsize*, *n*_*tree*_ and *m*_*try*_ by performing pairwise t-tests with a Benjamini-Hochberg adjustment. AUC scores were grouped by parameter values as indicated by a unique colour (orange for *sampsize*, blue for *n*_*tree*_ and pink for *m*_*try*_), resulting in 10 groups for *sampsize* (*n* = 150), 10 groups for *n*_*tree*_ (*n* = 150) and 15 groups for *m*_*try*_ (*n* = 100). A horizontal line is present in each plot, indicating the median of the lowest parameter value. Parameter values for *sampsize* were not found to differ significantly from each other, whereas, *n*_*tree*_ = 10 differed significantly from every other group and all *m*_*try*_ values demonstrated a difference with at least one other group. These findings suggest that lower *n*_*tree*_ values were associated with lower classification accuracy, with an opposite trend observed in the *m*_*try*_ parameter, where higher values were negatively correlated with classification accuracy. (TIFF 1373 kb) Additional file 6: Pairwise *t*-test results for *m*_*try*_ intra-parameter groups for the low *p/n* data. All *p*-values were adjusted using a Benjamini-Hochberg procedure. Each *sampsize* group was found to differ from at least 12 other groups. (TXT 2 kb) Additional file 7: Performance results are correlated between non-cross-validation results, 10-fold cross-validation and stratified 10-fold cross-validation. Correlations between non-cross-validation and cross-validation results of fitted random forest models to perform feature selection. (a) Non-cross-validation results were correlated to 10-fold cross-validation results (*ρ* = 0.084, *p* < 0.01, *ρ*_*c*_ = 3.9 × 10^−4^). (b) Non-cross-validation results were also correlated to stratified 10-fold cross-validation results (*ρ* = 0.1, *p* < 10^−4^, *ρ*_*c*_ = 2.9 × 10^−4^). (c) A very strong correlation was observed between stratified 10-fold cross-validation and 10-fold cross-validation (*ρ* = 0.65, *p* < 10^−179^, *ρ*_*c*_ = 0.63) with minimum AUCs of 0.9967 and 0.9952, respectively and 97 % of models overlapping at an AUC of 1. (TIFF 5780 kb) Additional file 8: AUC results for high *p/n* data. Validation results for all high *p/n* models (*n* = 1000) using the MSKCC data, DFCI data, and combined MSKCC and DFCI data. The AUC results and ranks are provided for each combination of *n*_*tree*_, *m*_*try*_ and *sampsize* parameters. Lower ranks represent higher model performance with 1 representing the most accurate model and 1000 representing the worst performing model. Logical columns are present to indicate whether a parameter set performed better than the default or well across all validation sets. Model performance was defined as good if the parameter set resulted in an AUC of > 0.6 across all validation sets. The default settings (*n*_*tree*_ = 500, *m*_*try*_ = 110, *sampsize* = 255) are found on row 596 of the table. (CSV 28 kb) Additional file 9: Intra-parameter values display variation for high *p/n* studies (combined validation data). The parameters *sampsize*, *n*_*tree*_ and *m*_*try*_ were analysed by performing pairwise t-tests with a Benjamini-Hochberg adjustment. AUC scores were grouped by parameter values as indicated by colour (orange for *sampsize*, blue for *n*_*tree*_ and pink for *m*_*try*_). In general, lower intra-parameter values for *sampsize*, *n*_*tree*_, and *m*_*try*_ were found to differ significantly from higher intra-parameter values, with higher parameter values exhibiting a positive correlation with AUC. (TIFF 1207 kb) Additional file 10: Pairwise *t*-test results for *n*_*tree*_ intra-parameter groups for the combined NSCLC validation data. All *p*-values were adjusted using a Benjamini-Hochberg procedure. Lower *n*_*tree*_ groups were found to differ from higher *n*_*tree*_ parameter groups for example, *n*_*tree*_ 10 from *n*_*tree*_ 10,000 − 500,000. (TXT 1019 bytes) Additional file 11: Pairwise *t*-test results for *m*_*try*_ intra-parameter groups for the combined NSCLC validation data. All *p*-values were adjusted using a Benjamini-Hochberg procedure. In general lower *m*_*try*_ values were found to differ significantly from higher *m*_*try*_ values for example, *m*_*try*_ 1 from *m*_*try*_ 110 − 2200. (TXT 1009 bytes) Additional file 12: Pairwise *t*-test results for *sampsize* intra-parameter groups for the combined NSCLC validation data. All *p*-values were adjusted using a Benjamini-Hochberg procedure. Significant differences were observed between large and small *sampsize* values, in particular, *sampsize* 26 from *sampsize* 102 − 255; *sampsize* 51 from *sampsize* 128 − 255; *sampsize* 77 from *sampsize* 128 − 255, etc. (TXT 1 kb) Additional file 13: AUC performance can be predicted for low *p/n* data using parameters as variables. Prediction accuracy (AUC) using the random forest classifier for low *p/n* data with Gini importance measures. (a) The model for the SeqControl data shows a strong correlation between predicted and observed AUC scores (*ρ* = 0.92, *p* < 10^−208^) and a Lin’s concordance correlation coefficient (*ρ*_*c*_) value of 0.89. (b) The Gini importance measures for the low *p/n* AUC values show that *m*_*try*_ is the most informative variable followed by *n*_*tree*_ and *sampsize*. (TIFF 80 kb) Additional file 14: AUC performance can be predicted for high *p/n* data using parameters as variables. Prediction accuracy using the random forest classifier for high *p/n* validation data with Gini importance measures. (a) The combined validation data demonstrated a strong correlation between the predicted and observed AUC values (*ρ* = 0.48, *p* < 10^−20^) and a *ρ*_*c*_ value of 0.33. (b) The relative order of Gini importance for the combined data was *sampsize* followed by *m*_*try*_ and lastly, *n*_*tree*_. (TIFF 68 kb) Additional file 15: Variable importance ranks according to *ntree* value. The ranks for the default parameters with unique *n*_*tree*_ values as column heads and sequencing quality metrics as row heads. The ranks stabilize at *n*_*tree*_ = 10,000. Using this criteria, the variable identified as the most important was “Average reads/starts” in 40 % of samples, whereas, [6] identified “% bases ≥ 8×” as the most important variable using the cforest algorithm. Below *n*_*tree*_ 1,000, “% bases ≥ 8×” was ranked as the most important variable in 40 % of samples. Although greater *n*_*tree*_ values may lead to more consistent rankings for variable importance, these values may become more biased through sampling with replacement methods [20]. (CSV 667 bytes) Additional file 16: Random forest usage in papers. A summary table of papers referencing random forest over a seven month period (January 1 to November 4) from BMC Bioinformatics. Information was recorded whether the paper uses a RF algorithm, and if so, whether they parameterized and report the tuned parameters. Eleven of sixteen papers use the RF algorithm and less than half of samples performed model tuning. An even fewer number of papers reported the optimized values [62–87]. (CSV 434 bytes) Additional file 17: Parameter grid for predicting performance. A summary table of parameters that were used to perform model tuning for predicting AUC using a subset of parameters, *n*_*tree*_, *m*_*try*_ and *sampsize*. A total of 162 parameters were used in model tuning and the optimal parameters (*n*_*tree*_ = 200, *m*_*try*_ = 2, *sampsize* = 200) were selected to fit the final model. (CSV 124 bytes)

## Abbreviations

AUC: Area under the receiver operating characteristic curve
DFCI: Dana-Farber Cancer Institute
HLM: Moffitt Cancer Center
HPCI: High performance computing interface
ICGC: International Cancer Genome Consortium
ML: Machine learning
MSKCC: Memorial Sloan-Kettering Cancer Center
NSCLC: Non-small cell lung cancer
OOB: Out-of-bag
RF: Random forest
RMSE: Root mean squared error
UM: University of Michigan Cancer Center
VIM: Variable importance measure
