# Supplementary figures and images for: The parameter sensitivity of random forests
Source: BMC Bioinformatics. 2016 Sep 1;17(1):331. doi: 10.1186/s12859-016-1228-x (PMC5009551; doi:10.1186/s12859-016-1228-x)

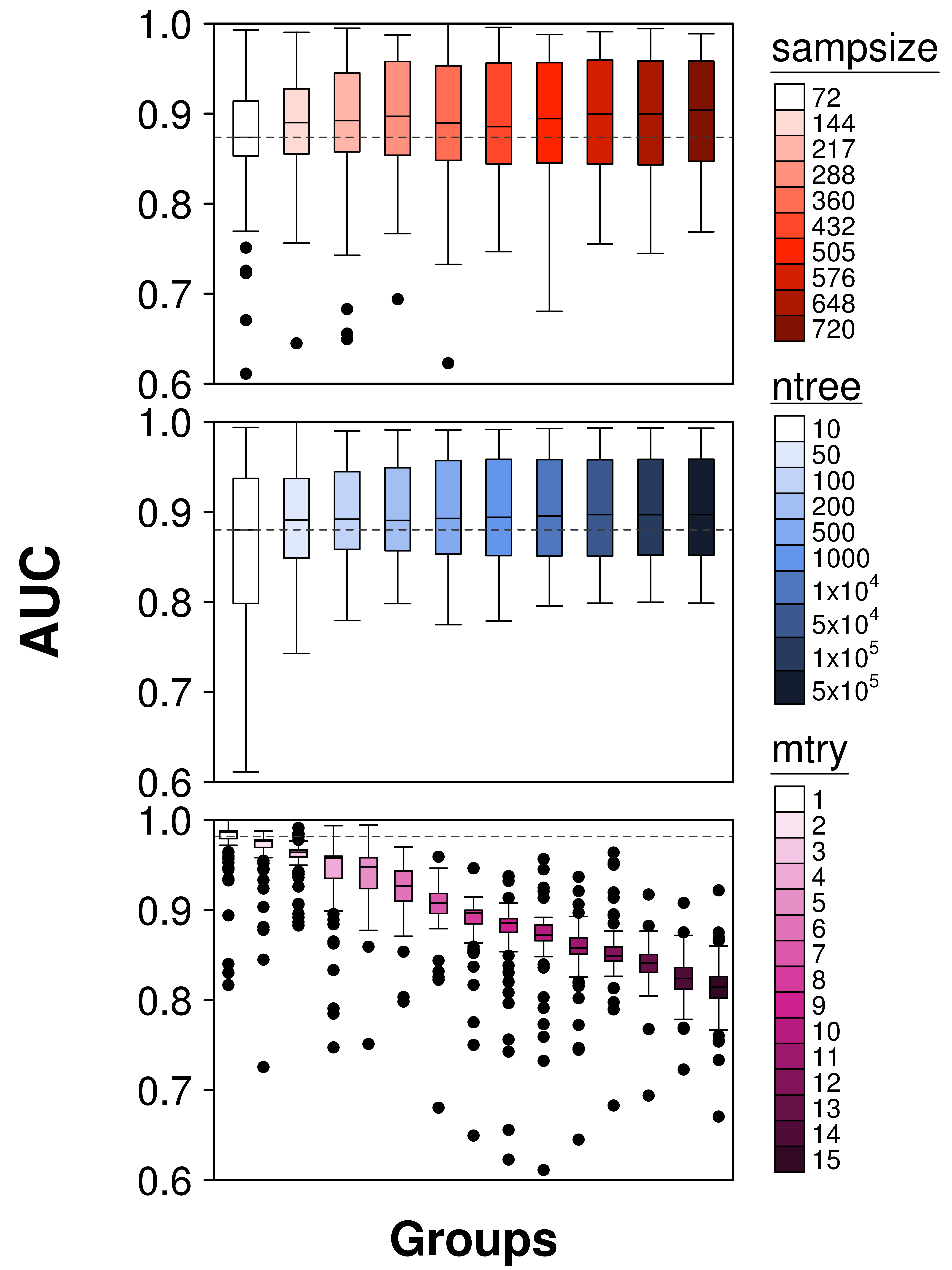

Supplement: Additional file 5: — Intra-parameter values display variation in low p/n studies. We evaluated the parameters sampsize, n tree and m try by performing pairwise t-tests with a Benjamini-Hochberg adjustment. AUC scores were grouped by parameter values as indicated by a unique colour (orange for sampsize, blue for n tree and pink for m try), resulting in 10 groups for sampsize (n = 150), 10 groups for n tree (n = 150) and 15 groups for m try (n = 100). A horizontal line is present in each plot, indicating the median of the lowest parameter value. Parameter values for sampsize were not found to differ significantly from each other, whereas, n tree = 10 differed significantly from every other group and all m try values demonstrated a difference with at least one other group. These findings suggest that lower n tree values were associated with lower classification accuracy, with an opposite trend observed in the m try parameter, where higher values were negatively correlated with classification accuracy. (TIFF 1373 kb) [file 12859_2016_1228_MOESM5_ESM.tiff]

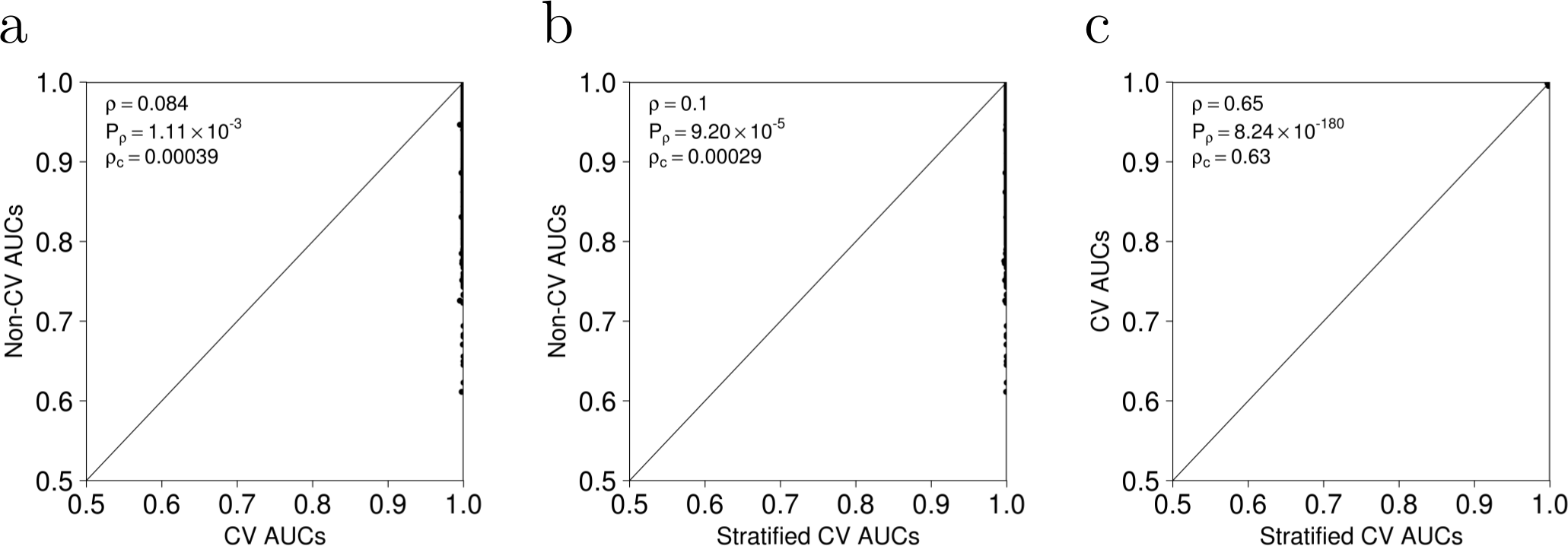

Supplement: Additional file 7: — Performance results are correlated between non-cross-validation results, 10-fold cross-validation and stratified 10-fold cross-validation. Correlations between non-cross-validation and cross-validation results of fitted random forest models to perform feature selection. (a) Non-cross-validation results were correlated to 10-fold cross-validation results (ρ = 0.084, p < 0.01, ρ c = 3.9 × 10^−4^). (b) Non-cross-validation results were also correlated to stratified 10-fold cross-validation results (ρ = 0.1, p < 10−4, ρ c = 2.9 × 10^−4^). (c) A very strong correlation was observed between stratified 10-fold cross-validation and 10-fold cross-validation (ρ = 0.65, p < 10−179, ρ c = 0.63) with minimum AUCs of 0.9967 and 0.9952, respectively and 97 % of models overlapping at an AUC of 1. (TIFF 5780 kb) [file 12859_2016_1228_MOESM7_ESM.tiff]

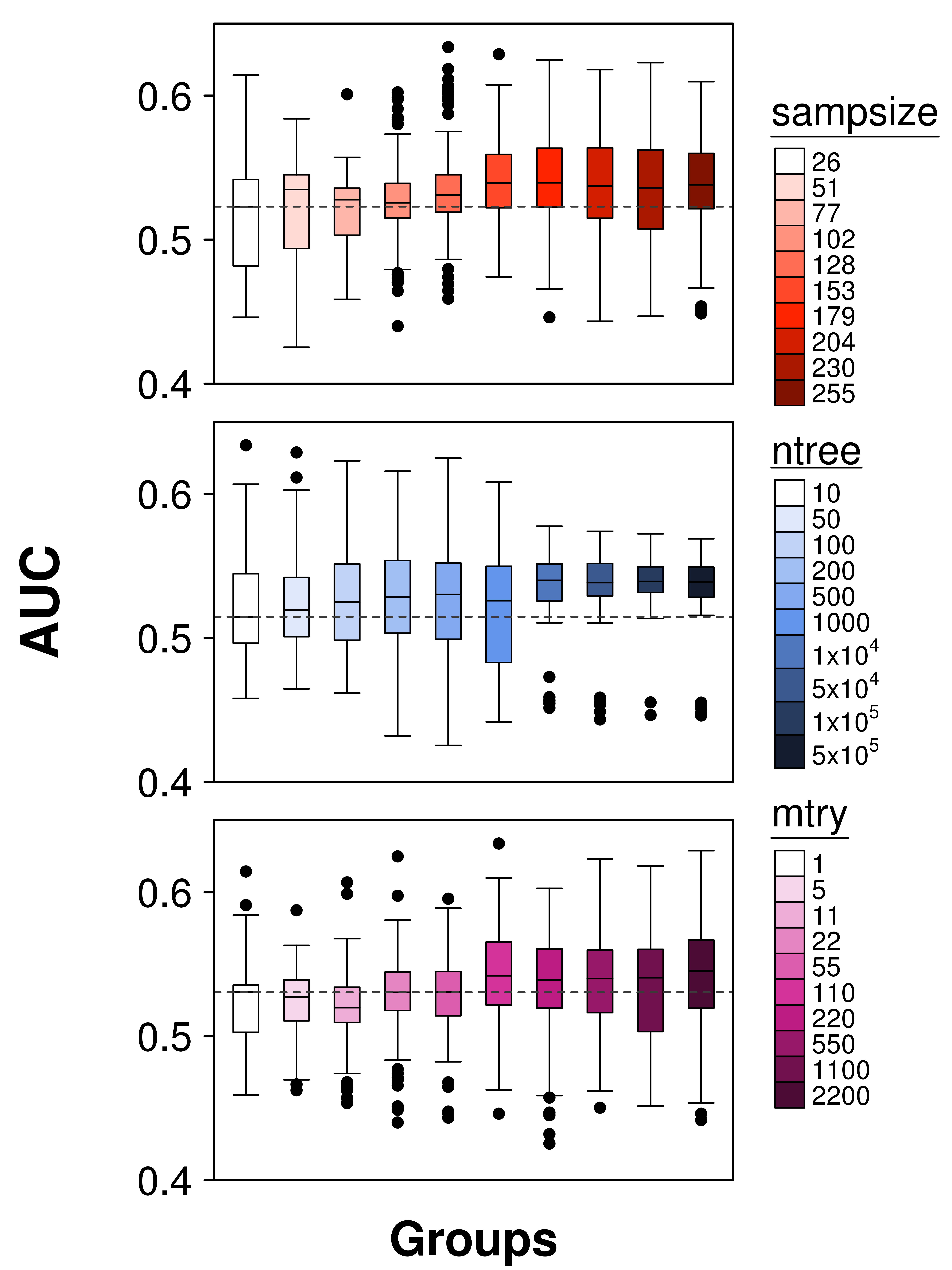

Supplement: Additional file 9: — Intra-parameter values display variation for high p/n studies (combined validation data). The parameters sampsize, n tree and m try were analysed by performing pairwise t-tests with a Benjamini-Hochberg adjustment. AUC scores were grouped by parameter values as indicated by colour (orange for sampsize, blue for n tree and pink for m try). In general, lower intra-parameter values for sampsize, n tree, and m try were found to differ significantly from higher intra-parameter values, with higher parameter values exhibiting a positive correlation with AUC. (TIFF 1207 kb) [file 12859_2016_1228_MOESM9_ESM.tiff]

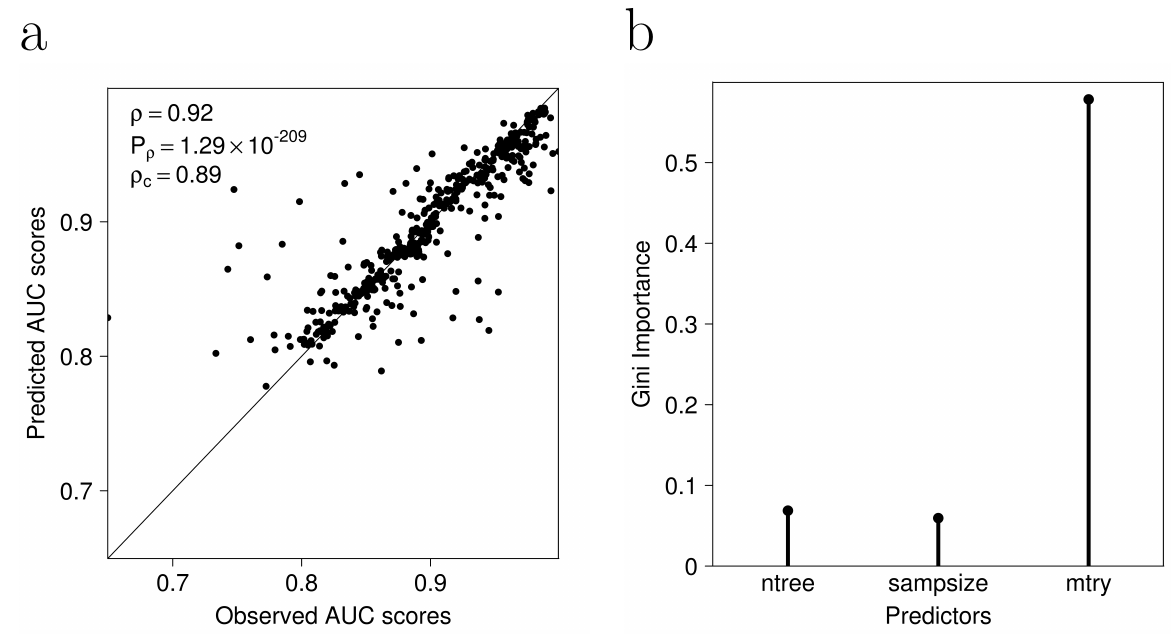

Supplement: Additional file 13: — AUC performance can be predicted for low p/n data using parameters as variables. Prediction accuracy (AUC) using the random forest classifier for low p/n data with Gini importance measures. (a) The model for the SeqControl data shows a strong correlation between predicted and observed AUC scores (ρ = 0.92, p < 10−208) and a Lin’s concordance correlation coefficient (ρ c) value of 0.89. (b) The Gini importance measures for the low p/n AUC values show that m try is the most informative variable followed by n tree and sampsize. (TIFF 80 kb) [file 12859_2016_1228_MOESM13_ESM.tiff]
